# Endoscopic Management of Complex Colorectal Polyps: Current Insights and Future Trends

**DOI:** 10.3389/fmed.2021.728704

**Published:** 2022-01-20

**Authors:** Rupinder Mann, Mahesh Gajendran, Chandraprakash Umapathy, Abhilash Perisetti, Hemant Goyal, Shreyas Saligram, Juan Echavarria

**Affiliations:** ^1^Department of Internal Medicine, Saint Agnes Medical Center, Fresno, CA, United States; ^2^Paul L. Foster School of Medicine, Texas Tech University Health Sciences Center, El Paso, TX, United States; ^3^Division of Gastroenterology, Long School of Medicine, University of Texas Health San Antonio, San Antonio, TX, United States; ^4^Department of Gastroenterology and Hepatology, The University of Arkansas for Medical Sciences, Little Rock, AR, United States; ^5^Interventional Oncology and Surgical Endoscopy (IOSE), Parkview Health, Fort Wayne, IN, United States; ^6^The Wright Center for Graduate Medical Education, Scranton, PA, United States

**Keywords:** colorectal polyp, colorectal cancer, endoscopic mucosal resection, endoscopic submucosal dissection, colonoscopy

## Abstract

Most colorectal cancers arise from adenomatous polyps and sessile serrated lesions. Screening colonoscopy and therapeutic polypectomy can potentially reduce colorectal cancer burden by early detection and removal of these polyps, thus decreasing colorectal cancer incidence and mortality. Most endoscopists are skilled in detecting and removing the vast majority of polyps endoscopically during a routine colonoscopy. Polyps can be considered “complex” based on size, location, morphology, underlying scar tissue, which are not amenable to removal by conventional endoscopic polypectomy techniques. They are technically more challenging to resect and carry an increased risk of complications. Most of these polyps were used to be managed by surgical intervention in the past. Rapid advancement in endoscopic resection techniques has led to a decreasing role of surgery in managing these complex polyps. These endoscopic resection techniques do require an expert in the field and advanced equipment to perform the procedure. In this review, we discuss various advanced endoscopic techniques for the management of complex polyps.

## Introduction

Colorectal cancer (CRC) is the third most common cancer diagnosed in both men and women in the United States each year (1, 2). In 2020, it was estimated that 149,500 adults were diagnosed with CRC. In terms of mortality, CRC ranks second as a cause of cancer mortality in both men and women combined, accounting for ~53,200 deaths in 2020 (2). The modifiable risk factors in CRC include smoking, high alcohol consumption, unhealthy diet, physical inactivity, and excessive weight attributing to more than half of cases of CRC (2). Most cases are preventable by appropriate screening and surveillance (3, 4).

The adenoma to carcinoma sequence is a well-established phenomenon in which normal colonic epithelium undergoes a series of genetic mutations that lead to cytological dysplasia and cancer (5, 6). The pathogenesis of genetic instability in CRC involves three major pathways: chromosomal instability (CIN), microsatellite instability (MSI), and CpG island methylator phenotype (CIMP) pathways (7). It is a slow process, usually takes 10–20 years, allowing effective detection of these polyps by screening colonoscopy (8). This sequence can be interrupted by polypectomy, thus decreasing the incidence and mortality from CRC (9–11). Although majority of CRC (70%) arises from adenomatous polyps, in about 25–30% of the cases, CRC develops from sessile serrated lesions (SSL) through the SSL-to-carcinoma pathway, mostly from the right colon. Most of the current literature on colon polyp progression to cancer is based on adenoma-carcinoma sequence, and thus in review, most of the information is inclined toward adenomatous polypectomy removal. Further changes will likely be seen in the future as more data emerges on the SSL to cancer pathways (12, 13).

The key variable in CRC prevention is polypectomy. There is no data from randomized controlled trials (RCT) to determine the effect of polypectomy on CRC incidence and mortality. The National Polyp Study is a pivotal study which provided strong evidence that polypectomy prevents CRC (9). In the National Polyp Study, 1,418 patients were included who had at least one adenoma resected during the colonoscopy and they were followed for a mean of 6 years. The incidence of CRC in the study cohort was significantly lower (76%) than expected on the basis of the rate in the Surveillance Epidemiology and End Results group. Furthermore, no CRC deaths were reported. In the long-term National Polyp Study follow-up study of 2,602 patients, the CRC mortality was reduced by 53% (95% CI 20–74%), when compared to the Surveillance Epidemiology and End Results population when followed for 23 years after polypectomy (10). A population based study from Germany showed that colonoscopy and polypectomy resulted in decreased CRC incidence and mortality, 10 years after the inclusion of colonoscopy to the national cancer screening program (14). There are three ongoing European Polyp Surveillance (EPoS) studies investigating the optimal surveillance strategies following adenoma and serrated polyp removal. EPOS I and II are randomized controlled trials, and EPOS III is observational. In EPOS I, 13,766 patients with low-risk adenomas (1–2 tubular adenomas of size <10 mm with low-grade dysplasia) are randomized to surveillance after 5 and 10 years or 10 years only. In EPOS II, 13,704 patients with high-risk adenomas (3–10 adenomas or adenomas ≥10 mm or with high-grade dysplasia or >25% villous features) are randomized to surveillance after 3, 5, and 10 years or 5 and 10 years only. EPOS III is an observational study where patients with serrated polyps ≥10 mm at any colorectal location or serrated polyps ≥5 mm proximal to the splenic flexure will undergo surveillance colonoscopy, 5 and 10 years after baseline colonoscopy. The primary endpoint of EPoS trials is the incidence of CRC, and it will be compared in all three different arms. This is the first long-term randomized trial to address surveillance after colorectal polyp removal (15).

More than 90% of polyps detected during screening colonoscopies are small (<10 mm in size), mostly benign, and do not contain advanced disease. These can be easily managed by conventional cold forceps or by snare polypectomy (12, 16–18). Around 10–15% of colorectal polyps are considered “complex” as they are difficult to be appropriately removed with these conventional endoscopic methods due to their size, location, and morphology. This review aims to discuss complex polyps and provides in depth overview of different endoscopic methods for removing these complex polyps. We also discuss various complications associated with these procedures and also future directions in the field.

## Complex Polyp

Complex colon polyps are generally characterized as any lesion whose endoscopic resection is technically challenging due to the size (>20 mm), the shape (flat/bulky), extent (polyps crossing two haustral folds, and polyps occupying more than a third of lumen circumference), location (right side, ileocecal valve, dentate line), or due to the presence of fibrosis as a consequence of large laterally spreading lesions (LSL) or previous attempts of endoscopic resection (ER) (19–25). These complex polyps carry an increased risk of colorectal cancer, high recurrence rates in the range of 10–20% after piecemeal resection, risk of adverse events with resection, increased risk of interval cancer after incomplete resection, and potential for increased medicolegal risks (26, 27).

Approximately 10% of polyps are incompletely resected, mainly due to size and morphology, which might contribute to interval cancer (28). Most large polyps can be effectively and safely resected by advanced endoscopic techniques, such as endoscopic mucosal resection (EMR) and submucosal endoscopic dissection (ESD) (12). These techniques are usually indicated when polyps are confined to the colonic mucosa (epithelium, lamina propria, and muscularis mucosa), an area where there is no lymphatic drainage, and the risk of lymph node metastasis (LNM) is extremely low (29). Selected superficially invasive cancers can also be resected by en-bloc EMR or ESD. Endoscopic resection of unrecognized malignant polyps with superficial submucosal invasive cancer (SMIC), with subsequent surgical resection, is not associated with increased risk of lymph node metastasis recurrence or decreased long-term recurrence-free survival, even with high-risk histologic features (30, 31).

Malignant polyps, those which invade the submucosa (submucosal invasive cancer–SMIC) but do not extend into the muscularis propria (T1 on TNM classification), have a prevalence of about 0.2–5% (32). In large, non-pedunculated polyps, SMIC is seen in about 15% of polyps, with less than half having deep submucosal invasion (33).

### Classification of Polyps

**Figure 1:**
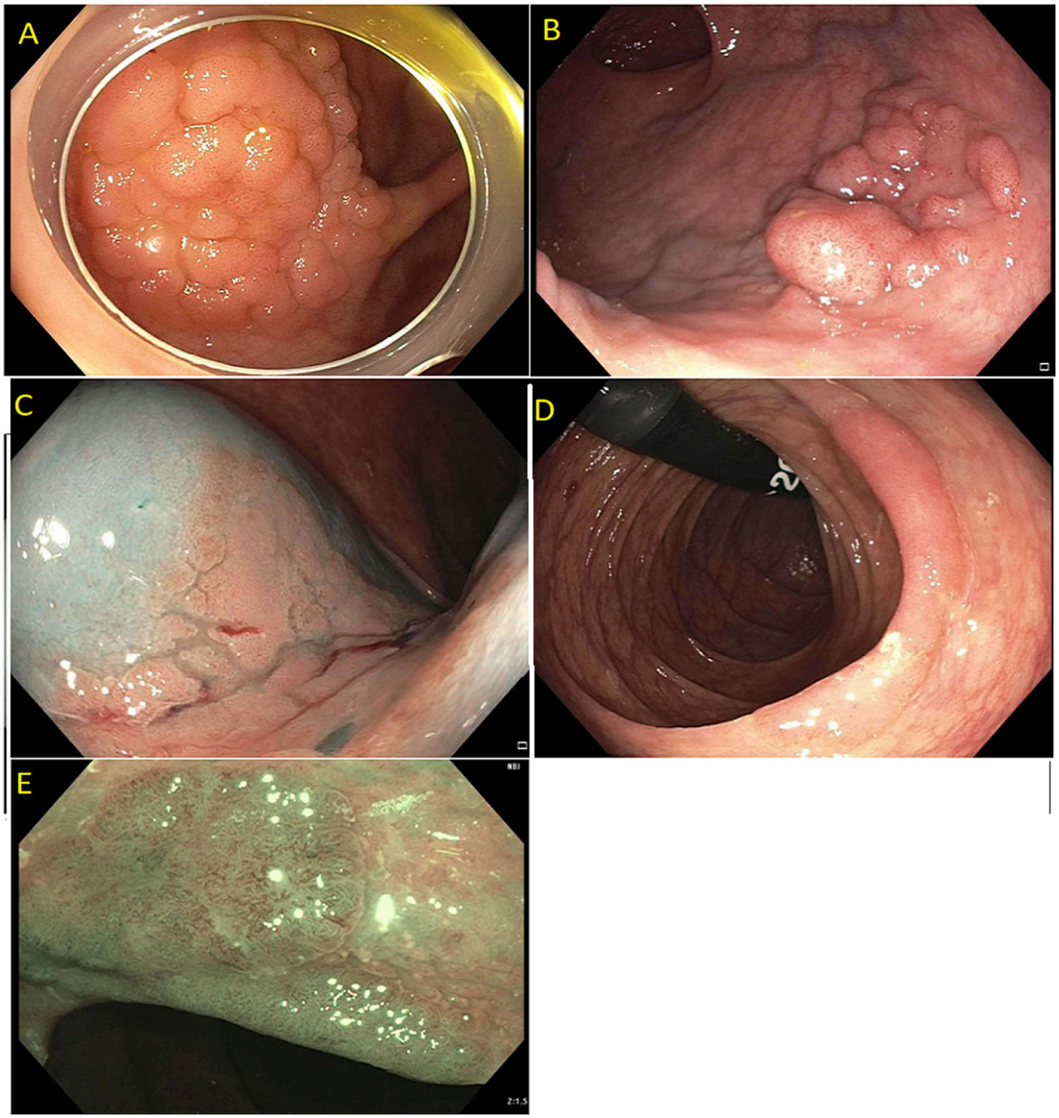
**(A)** Granular lateral spreading lesion; **(B)** Granular lateral spreading lesion with dominant nodule; **(C)** Non-granular lateral spreading lesion. These lesion have a higher risk of fibrosis and invasive cancer. Polyp was tubular adenoma; **(D)** Non-granular lateral spreading lesion on white light; **(E)** Non-granular lateral spreading lesion on Narrow Band Imaging (NBI). Histology revealed a T1 adenocarcinoma.

**Figure 2:**
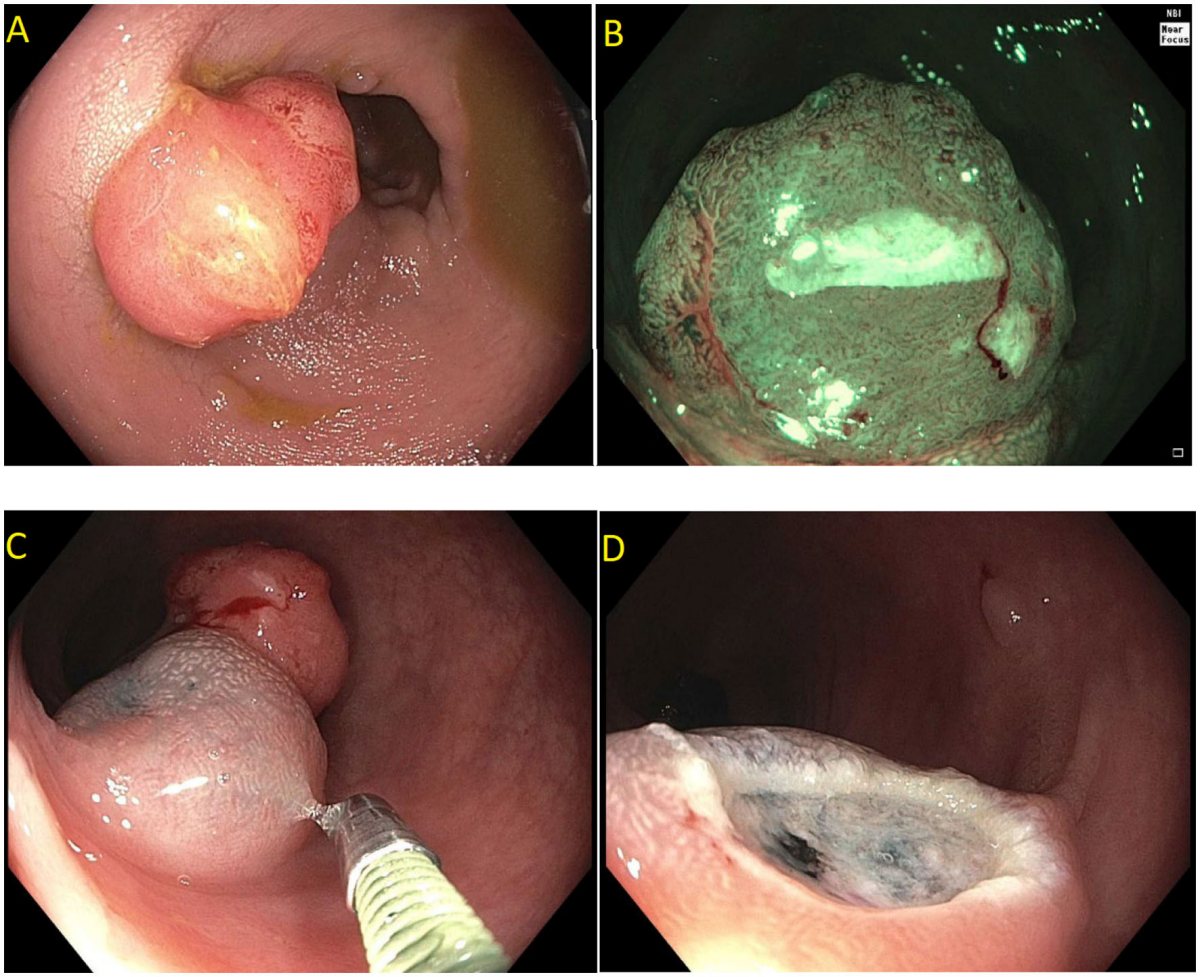
Paris is lesion in the rectum. **(A)** Seen on white light; **(B)** Seen on Narrow Band Imaging (NBI); **(C,D)** Polyp raised and resected en bloc. Histology revealed a superficial (<1 mm) T1 tumor with lymphovascular invasion.

Detailed endoscopic assessment of a lesion with high-definition imaging is a critical first step for the optimal management of colorectal polyps. However, high-definition white light evaluation alone for features such as fold convergence, edge retraction, expansion/thickened folds, firm consistency, erythema is not enough for an assessment of SMIC. Increasing size, recto-sigmoid location, and surface morphology have been associated with an increased risk of SMIC (34–36). Lateral spreading lesions (LSL), polyps that spread laterally and circumferentially rather than vertically, are commonly seen in practice (Figure 1). These lesions can be large and technically challenging to remove due to size, location, and fibrosis. The frequency of invasive cancer in homogeneous granular lateral spreading lesions (G-LSL) tends to be lower (<5%) than for G-LSL with a dominant nodule and for non-granular LSL (NG-LSL), which are flat or pseudo-depressed, as well as large sessile and bulky lesions of similar size (Figure 2) (35, 37).

**Figure 3:**
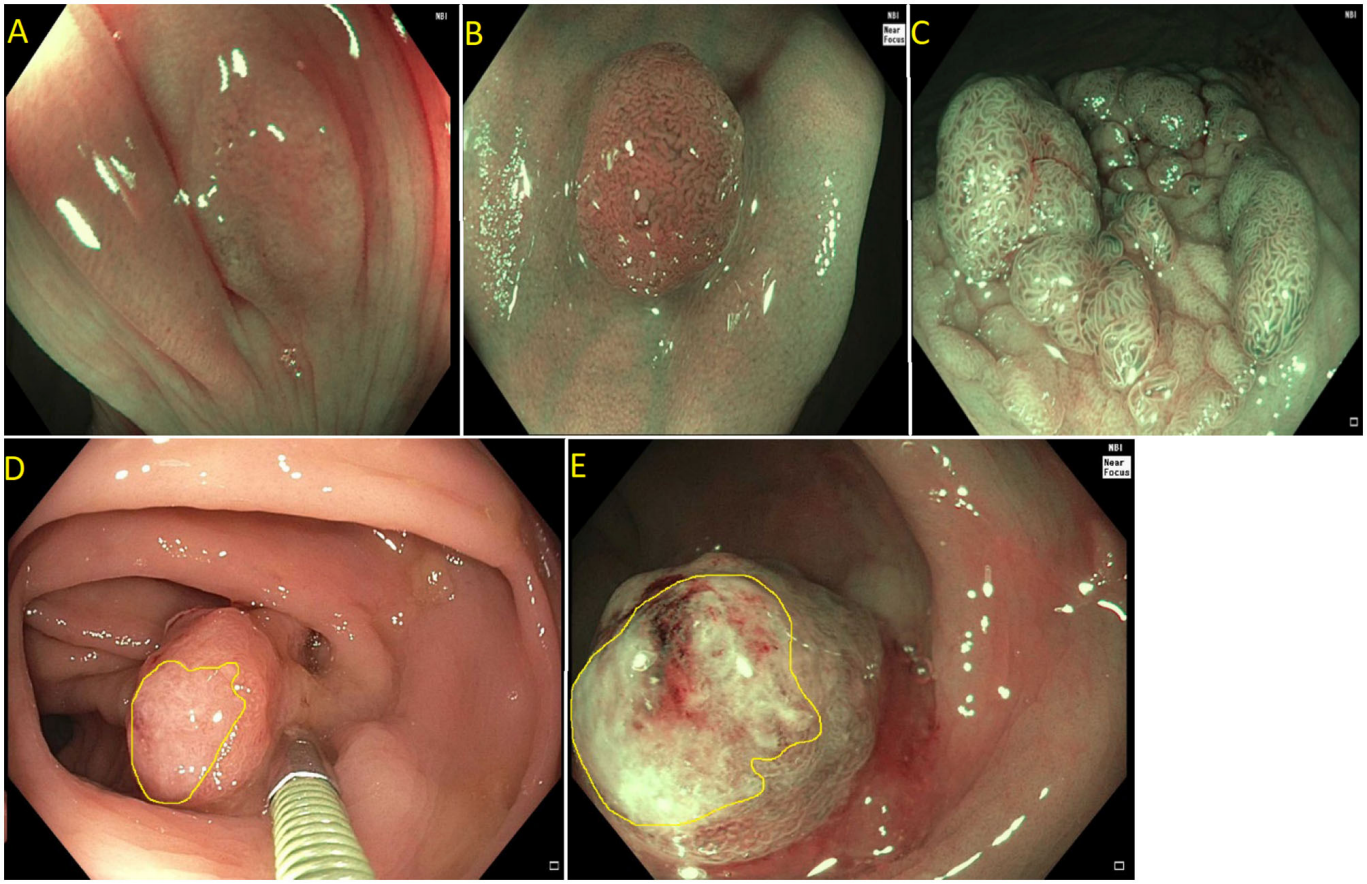
**(A)** NICE type I (hyperplastic polyp); **(B)** Paris I-s, NICE type II (tubular adenoma without high grade dysplasia); **(C)** Paris IIa + is lateral spreading lesion, NICE type II (tubulovillous adenoma without high grade dysplasia); **(D)** NICE type III (adenocarcinoma) as see on white light. Note the invisible surface pattern with avascular area, highlighted in yellow; **(E)** NICE type III (Adenocarcinoma) as see under NBI.

**Figure 4:**
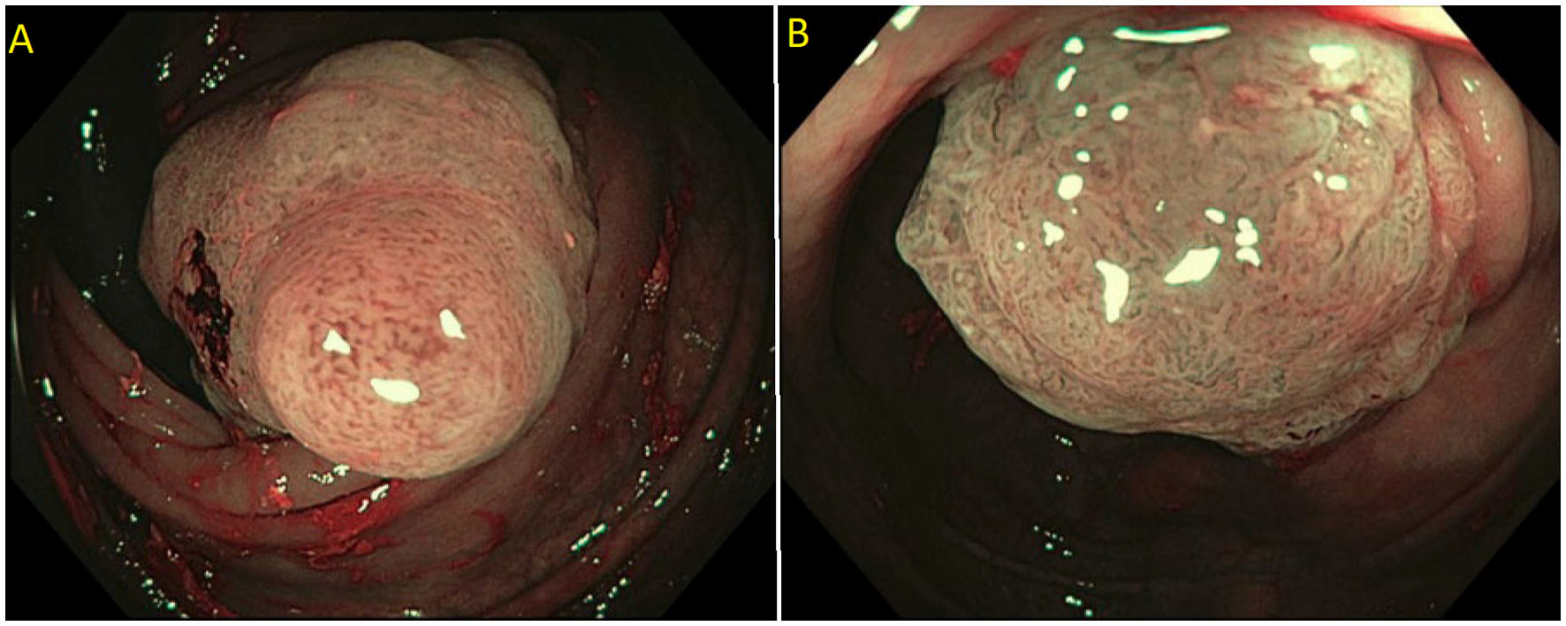
**(A)** Paris 0-IIa lateral spreading lesion; **(B)** On NBI, lesion classified as a JNET 2B. Histology revealed tubular adenoma with high grade dysplasia.

**Figure 5:**
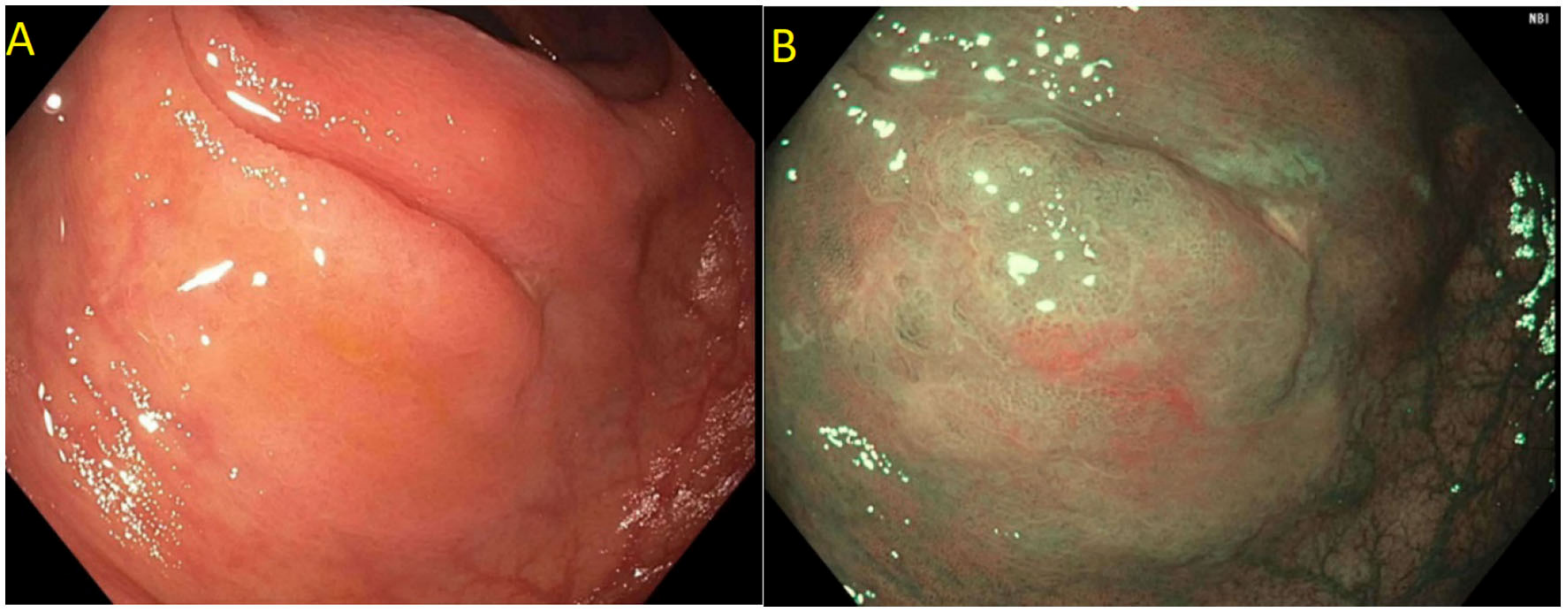
Sessile serrated polyp on white light **(A)** and narrow band imaging **(B)**. Polyp lacks a brown coloration and blood vessels or a tubular/branched surface pattern seen with tubular adenomas. Features of SSPs include clouded surface, indisctinctive borders, irregular shape, dark spots inside crypts, and mucus cap.

Current US Multi-Society Task Force guidelines recommend endoscopic lesion assessment by using aids such as the Paris classification, virtual chromoendoscopy (such as Narrow Band Imaging, or dye spray chromoendoscopy (Kudo classification) for detection of features suggestive of deep SMI. The Paris classification is a morphological classification of polyps that can predict invasive disease risk in lesions (38). Based on the Paris classification, polyps can be classified as protruding (0-Is—sessile, 0-Ip—pedunculated, and Isp—semi-pedunculated), flat (elevated 0-IIa, flat 0-IIb, and depressed 0-IIc) and excavated (Type 0-III). The type 0-III lesions are uncommon in the colon. Depressed lesions have an increased risk of malignancy (30–50% of cases). Combining Paris classification and the LSL classification can help guide risk of SMIC. Endoscopic assessment of surface characteristic can be assisted by “real-time” manipulation of wavelengths that enhance blood vessels and delineate surface features [e.g., narrow band imaging (NBI); Olympus, Center Valley, PA and Fujinon Blue Light Imaging; Fujinon, Valhalla, NY] or by postprocessor technologies that recreate the image as per the desired wavelengths (e.g., Fujinon Linked Color Imaging and Pentax iscan; Pentax Medical, Montvale, NJ) (32, 39). The Narrow Band Imaging International Colorectal Endoscopic (NICE) classification allows examination of the surface characteristic of a polyp based on surface appearance, color and vessel pattern. The NICE classification is highly accurate in classifying polyps into type 1 (hyperplastic), type 2 (adenoma), and type 3 (invasive cancer) (Figure 3) (40–43). For the latter, the NICE criteria carry a high specificity but low sensitivity. In order to overcome this limitation, the Japanese Narrow Band Imaging Expert Team (JNET) further divides type 2 into JNET 2a (conventional adenoma) and JNET 2b (adenoma with high grade dysplasia or superficial SMIC) (Figure 4) (44). The WASP criteria, based also on NBI findings, was developed to help identify sessile serrated lesions (Figure 5). A lesser used tool in the United States, the Kudo Pit Pattern Classification, uses a combination of magnifying colonoscopy with dye spray (Indigo Carmine and Cresyl Violet) to highlight the pit pattern and determine the risk of deep submucosal invasion (45). Malignant colorectal polyps are further divided based on the histopathological feature. The Kikuchi classification system describes submucosal invasion in sessile and flat malignant colorectal polyp by dividing submucosa into three levels: sm1 describes invasion into the upper third of submucosa, sm2 describes invasion into the middle third of submucosa and sm3 describes invasion into the lower third of submucosa. The penetration of cancer cells into sm3 is associated with a higher risk of lymphatic spread. This implementation of this classification is challenging as it depends upon the quality of resected specimen as the entire submucosa is not typically included in the specimen (46, 47). The Haggitt criteria, used mainly for pedunculated polyps, classifies polyps into 0–4 levels based on the depth of invasion. In level 0, dysplastic cells are limited to the mucosa, level 1 indicated invasion of cancer cells into submucosa but limited to head of polyp; level 2 indicates invasion of cancer cells into neck of the polyp; level 3 indicates when cancer cell invade stalk of the polyp, and level 4 indicate when cancer cells invade submucosa below stalk of polyp but above muscularis propria. All non-pedunculated polyps with any degree of submucosal indicate level 4. The higher depth of invasion is found to be associated higher incidence of lymph node invasion. Like the Kikuchi classification, this classification system also depends on the resected specimen's quality, as if a pedunculated polyp is resected through the stalk, it will limit the classification (48, 49).

According to the 2019 Japanese Society for Cancer of the Colon and Rectum (JSCCR) guidelines, early CRC (cT1) is further categorized into slightly invasive cT1 and deeply invasive cT1. Deeply invasive cT1 is defined based on the endoscopic findings such as fullness, erosion, ulcer, deformity, rigidity, and full convergence on white light; contrast imaging; dye chromoendoscopy or image enhanced endoscopy (e.g., NBI, BLI); and endoscopic ultrasound findings. Deeply invasive cT1 lesions are managed with surgical resection with varying degrees of lymph node dissection due to high risk of lymph node metastasis. Slightly invasive cT1 (cTis) can be managed with endoscopic treatment through EMR or ESD when en bloc resection is possible due to low risk of lymph node metastasis. Whenever en bloc resection is not possible, these lesions are managed surgically. Even when endoscopic resection is successful, tumors with unfavorable histological features need lymph nodes dissection. These include: positive vertical margin, deep invasion (T1b, submucosal invasion ≥1,000 μm), poorly differentiated adenocarcinoma, signet-ring cell carcinoma, or mucinous carcinoma, and budding grade of BD2/3 at the site of deepest invasion (50, 51).

As per the American Joint Committee on Cancer (AJCC), early colorectal lesions, including malignant colorectal polyps, are defined as cancer invading through the muscularis mucosa into the submucosa (T1). This is further subclassified into T1a when the lesion is restricted to muscularis mucosa and T1b when the lesion is extending to submucosa. In patients with T1a lesions with low-risk features (well-or moderately differentiated adenocarcinoma, resection margins free of dysplasia or cancer, ≤ 2 mm depth of submucosal invasion, absence of angiolymphatic invasion), endoscopic management with EMR or ESD is sufficient if en bloc resection with negative margins can be achieved. However, for the patients with high-risk lesions and or T1b (poorly differentiated adenocarcinoma, cribriform pattern, >2 mm depth of submucosal invasion, lymphatic invasion, and tumor budding) surgical resection with lymph node dissection is recommended since they have a risk of lymph node metastasis (52–54).

### Assessment of the Technical Difficulty

The second step in the resection of complex polyps is based on the assessment of the technical difficulty. It is well-recognized that incomplete resection is common, increases the difficulty for subsequent EMR or ESD, and is a risk factor for the need for surgical resection. The SMSA scoring system (size–S, morphology–M, site–S, and access–A) is a simple clinical score that helps to predict the difficulty in polypectomy and identify patients who are at increased risk of incomplete resection, adverse events, and recurrence based on the above-mentioned polyp characteristics (55–57).

Complex polyps should be managed by expert endoscopists with training in advanced polypectomy techniques in a multispecialty setting due to higher risk of complications like bleeding compared to conventional polypectomy; to minimize the risk of residual polyp/recurrence; to avoid unnecessary surgeries for benign polyps, and to achieve optimal oncologic resection in case of malignant polyps (26).

## Surgical Resection

It is extremely important to identify malignant polyps prior to endoscopic resection to provide the best outcomes, as polyps with deep submucosal invasion are best treated with surgical resection. However, many patients in the United States still undergo surgical resection for benign colon polyps, independent of age, race, sex, or ethnicity (58). In an analysis of a large, nationally representative sample, it was found that surgery for nonmalignant colorectal polyps has significantly increased from 5.9 in 2000 to 9.4 in 2014 per 100,000 adults (incidence rate difference, 3.56; 95% CI 3.40–3.72) (58). Unnecessary surgical management results in increased morbidity, mortality, and direct and indirect costs (59, 60). In a large multicenter study, endoscopic management of large LSL by EMR was significantly more cost-effective than surgery, with a mean cost saving of $7,602 per patient (95% CI: $8,458–$9,220) and a reduction of inpatient hospitalization length of stay by 2.81 nights per patient (95% CI: 2.69–2.94) (60). A prospective study from National Surgical Quality Improvement Program included 12,732 patients who underwent elective surgery to remove the non-malignant colorectal polyps. This study showed that the overall risk of 30-day mortality was 0.7%, and the risk of one or more major postoperative adverse events was 14%. The index surgery resulted in ostomy among 2.2% of the study population (61).

### Transanal Minimally Invasive Surgery

For the last 3 decades, trans-anal endoscopic microsurgery (TEM) has been the primary treatment for large, benign lesions of the rectum. However, the cost and technical complexity of the procedure limits its general use by colo-rectal surgeons. Transanal minimally invasive surgery (TAMIS) is a minimally invasive technique for resection of rectal tumors and was first described in 2009 by Atallah et al. as an alternative to TEM. EMR and ESD provide an endoscopic alternative for treating complex rectal lesions (62). There is very limited data comparing TAMIS with ESD.

In a single-center uncontrolled prospective study conducted in Germany, 330 patients referred for endoscopic resection of rectal large non-pedunculated colorectal polyps (LNPCPs) were included. ESD was performed in 302 patients with rectal LNPCPs, and the remaining 28 patients (advanced cancer was suspected macroscopically in 20 patients and benign lesion in 8 patients) were included. The resected lesion showed submucosal invasive cancer (SMIC) in 52 patients (17.2%) and benign lesions in 250 patients (82.8%). For SMIC, en bloc, R0, and curative resection were achieved in 81.4, 65.1, and 30.2% cases. Over the course of the study period, the curative resection rate increased from 13.6 to 47.6%, *p* = 0.036. En bloc and R0 resection for benign lesions was achieved in 83.2 and 70% cases, respectively. The total recurrence rate was seen in 4.8% cases for benign lesions after ESD (63). Quaresima et al. conducted a prospective study of 31 patients who underwent single-port TAMIS for mid and high rectal tumors. TAMIS was successfully completed in all cases without conversation into transabdominal surgery. The overall complication rate was 9.6%, including one case of urinary tract infection, one subcutaneous emphysema, and one hemorrhoidal thrombosis. R0 resection was allowed in 96.8% of cases with TAMIS. At a mean follow-up of 30 months, a single case of local recurrence occurred after large adenoma resection (64).

A multicenter randomized controlled trial (NL7083) is currently ongoing in Netherlands comparing TAMIS and ESD for the resection of non-pedunculated rectal lesions >2 cm size, with the bulk of lesion located below 15 cm from the anal verge (65). A target sample size is 198 patients who would be randomized into TAMIS and ESD arms. The primary endpoint is the recurrence rate at follow-up colonoscopy at 6 months. Secondary endpoints include radical (Ro-) resection rate, perceived burden and quality of life, cost-effectiveness, surgical referral rate, overall complication rate, and recurrence rate at 24 months (65).

## Complex Polypectomy

Although most of the complex polyps are benign, and >90% of these can be safely resected endoscopically, assessment of malignancy should be determined first, as deeply invasive cancer should be removed surgically for complete resection and histologic assessment of lymph nodes to determine lymph node metastasis. Visual signs suggestive of malignancy on colonoscopy evaluation include induration, friability, ulceration, and fixation to the colonic wall. However, large polyps can have invasive carcinoma without these signs (24, 66–70). Technique selection varies based on location, the lesion's morphology, patient's comorbidities, and endoscopist skills (71, 72). Advanced endoscopic techniques include EMR, ESD, hybrid techniques such as pre-cut EMR, Hybrid ESD, and novel therapies such as endoscopic full-thickness resection.

## Approach and Resection Techniques

### Endoscopic Mucosal Resection

**Figure 6:**
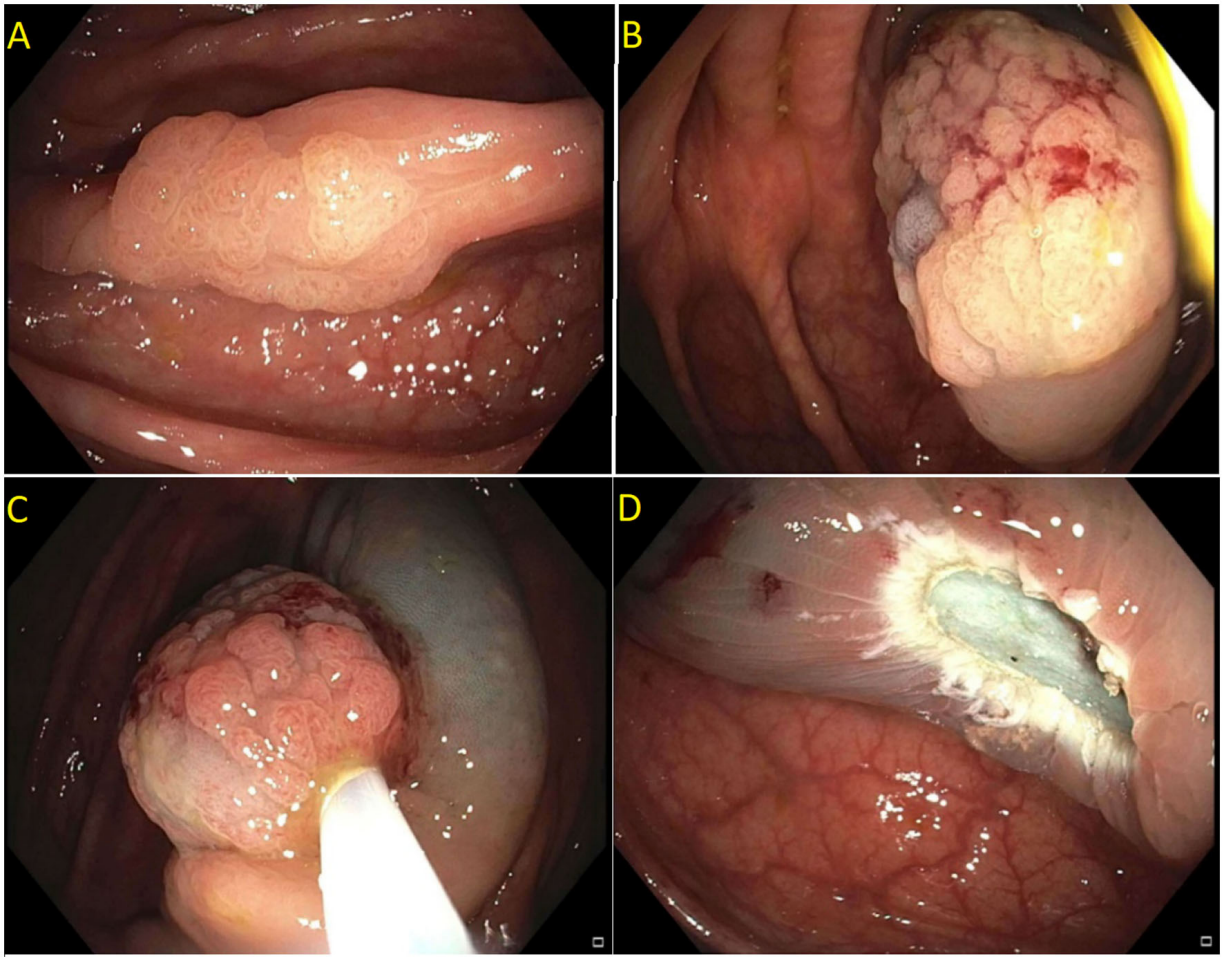
**(A,B)** Paris 0-IIa lesion, injected with methylene blue, size noted to be larger than originally suspected; **(C,D)** En-bloc endoscopic mucosal resection with blended coagulation current and a 20 mm snare.

**Figure 7:**
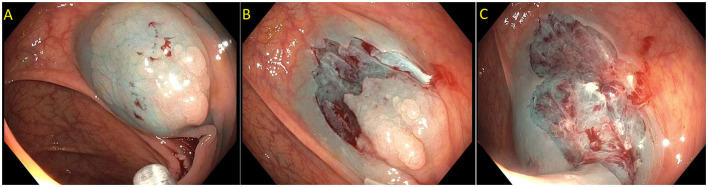
**(A)** Sessile serrated lesion injected prior to resection to better define resection borders; **(B,C)** Sessile serrated lesion removed by dynamic submucosal injection and piecemeal cold endoscopic mucosal resection.

**Figure 8:**
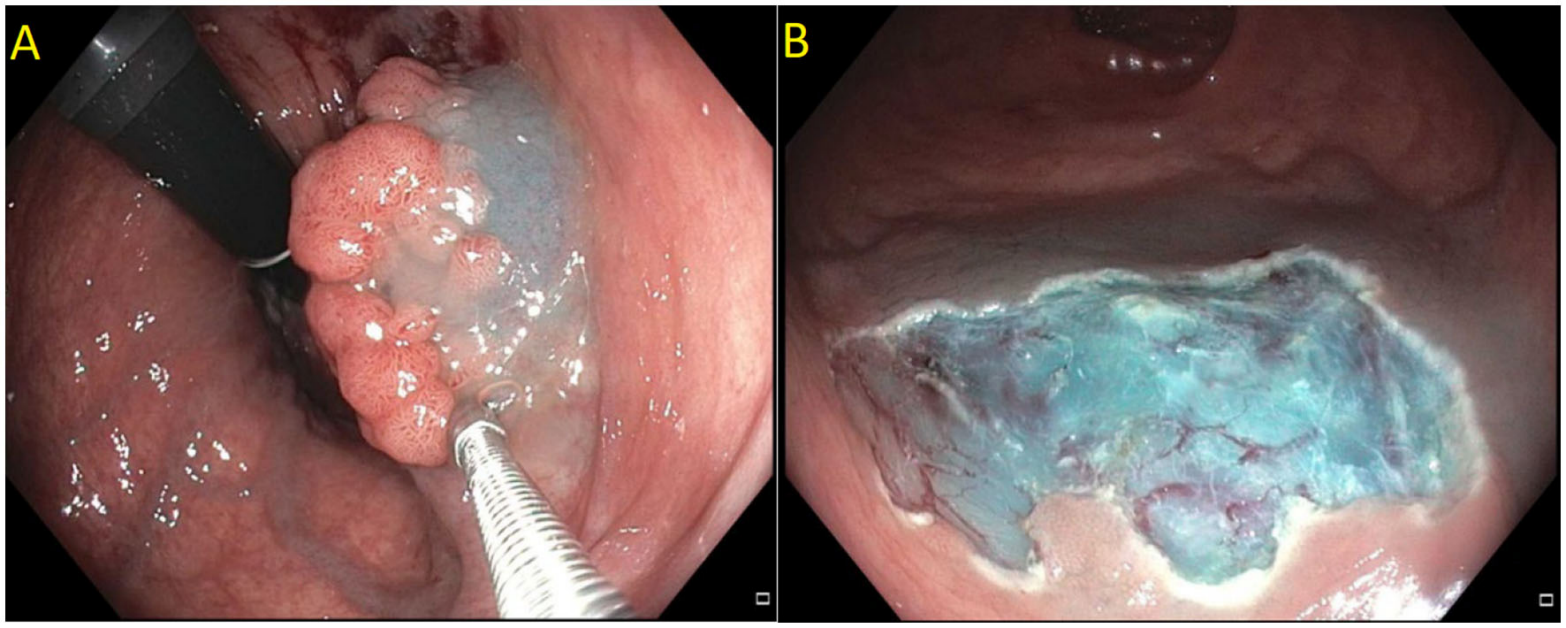
**(A)** Submucosal injection using ORISE™ gel submucosal lifting agent (Boston Scientific). **(B)** Submucosa easily identify with indigocarmine non-vital stain.

**Figure 9:**
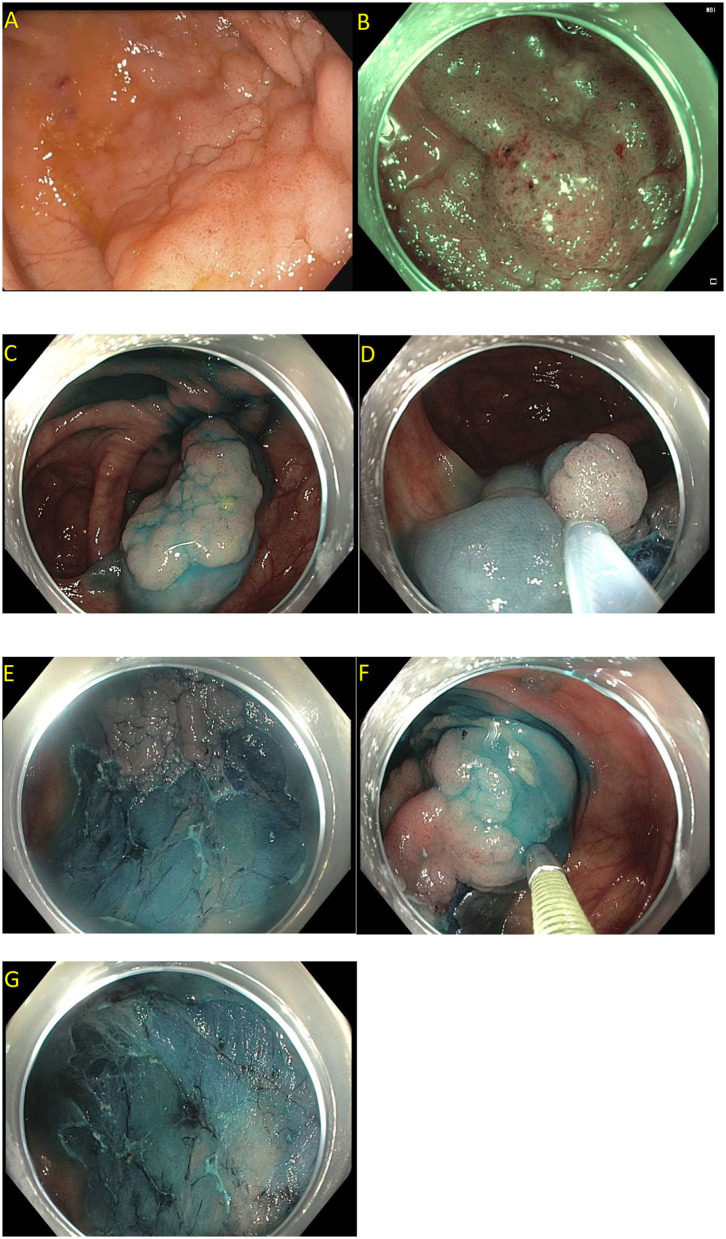
Piecemeal endoscopic mucosal resection. **(A)** A 40 mm Paris 0-IIa, granular lateral spreading lesion in the cecum seen on white light; **(B)** Same lesion seen under narrow band imaging; **(C–G)** Polyp removed by dynamic and piecemeal injection using a blended cutting current. The histology showed tubular adenoma.

Endoscopic mucosal resection (EMR) is a technique that involves the removal of lesions within the mucosa (71). EMR technique involves submucosal injection of a solution into submucosal space, thus lifting lesion away from the muscularis propria of the colon, followed by cautery snare resection (Figure 6) (12, 73). Cold snare EMR is a widely used technique for polyps sized <10 mm, with emerging data that supports its use for polyps between 10 and 20 mm and even beyond 20 mm, specially for serrated lesions (Figure 7) (18, 74–76). There are various solutions available for submucosal injections, with sterile normal saline being most frequently used. Other injectable solutions include saline with epinephrine, fibrin glue, hyaluronic acid, hydroxypropyl methylcellulose, succinylated gelatin and, glycerol (77–81). Vital dyes like methylene blue or non-vital dyes like indigo carmine help identify the deep muscular layer injury or perforation (82, 83). A meta-analysis of five randomized controlled studies showed a significant increase in en-bloc resection (OR 1.91, 95% Cl: 1.11–3.29, *P* = 0.02) and fewer residual lesions (OR 0.54, 95% Cl: 0.32–0.91, *p* = 0.02) with viscous solutions compared to normal saline used for submucosal injection for EMR (84). The US multi-society task force on colorectal cancer recommends the use of a viscous injection solution (e.g., hydroxyethyl starch, Eleview^®^ submucosal injectable composition, ORISE™ Gel Submucosal Lifting Agent, Boston Scientific) for lesions ≥20 mm to remove the lesion in a piecemeal fashion with less procedure time compared to normal saline (Figure 8). It also recommends the use of contrast agents, such as indigo carmine or methylene blue, in the submucosal injection solution to facilitate recognition of the submucosa from the mucosa and muscularis propria layers (85). Lesions are removed by snare excision either as en-bloc resection or piecemeal polypectomy, depending on size and morphology (Figure 9). A meta-analysis of 50 studies, including 6,442 patients with colorectal polyps ≥20 mm treated with EMR, showed an initial success rate of 92% for endoscopic resection, and only 8% of patients underwent surgery due to non-curative endoscopic resection. Endoscopic recurrence, perforation and bleeding occurred in 13.8, 1.5 and 6.5%, respectively (86). Studies have shown that EMR is not only cost-effective than surgery; it has less morbidity and mortality also (Table 1). It should be considered the first line of treatment for patients with these sessile or lateral spreading large (≥20 mm) lesions (60, 87).

**Table 1:** Endoscopic mucosal resection (EMR) for colon polyp studies with more than 100 patients.

**References**	**Study type**	**Number of patients**	**En bloc resection rate (%)**	**Piecemeal resection (%)**	**Complications (%)**
**2000–2010**					
Church (88)	Prospective	252	30	70	Late bleeding-6.74, perforation-0, post-polypectomy syndrome-0.79
Doniec et al. (89)	Prospective	184	11	89	Bleeding-2, perforation-0.5
Conio et al. (90)	Prospective	136	0	100	Intraprocedure bleeding-10.8, perforation-0
Perez Roldan et al. (91)	Retrospective	142	49	51	Bleeding-5.4, perforation-1.3
Uraoka et al. (92)	Retrospective	211	56.05	n/a	Immediate bleeding-4, delayed bleeding-4.9, perforation-0.4
Overhiser and Rex (93)	Retrospective	184	15	85	Delayed bleeding-7.3, perforation-1.1, post-polypectomy syndrome-0.6
Arebi et al. (94)	Retrospective	161	0	100	Bleeding-1.7, perforation-0
Swan et al. (95)	Prospective	174	33.53	66.1	Delayed bleeding-3.7, perforation-0
Khashab et al. (96)	Retrospective	132	0	100	Delayed bleeding-4.5, perforation-0
Luigiano et al. (97)	Retrospective	148	43.9	56.1	Procedural bleeding-10.14, perforation-0.68, post-polypectomy syndrome-1.35
Conio et al. (98)	Prospective	255	0	100	Intraprocedural bleeding-7.4, perforation-0, post-coagulation syndrome-0.3
Saito et al. (99)	Retrospective	228	33	67	Delayed bleeding-3.1, perforation-1.3
**2011–2020**					
Tajika et al. (100)	Retrospective	104	83.5	n/a	Bleeding-2.9, perforation-0
Buchner et al. (101)	Retrospective	274	53.5	46	Acute bleeding-3.38, delayed bleeding-7.2, microperforation-0.36
Kim et al. (102)	Retrospective	497	72.4	27.6	Procedural bleeding-18, post-EMR bleeding-2, perforation-0.4
Lee et al. (103)	Retrospective	140	42.9	57.1	Bleeding-0, perforation-0
Serrano et al. (104)	Retrospective	133	56.4	43.6	Intraprocedural bleeding-4.3, delayed bleeding-0.7, perforation-0.7
Belle et al. (105)	Retrospective	147	58	24	Bleeding-14, perforation-8.8
Bronsgeest et al. (106)	Retrospective	343	18.7	81.3	Bleeding-6.9, perforation-1.2
Pellise et al. (107)	Prospective	1,671	15.8	84.2	Bleeding-n/a, perforation-0.48
Zhang et al. (108)	Prospective	179	95	5	Bleeding-1.65, perforation-n/a
Iwashita et al. (109)	Retrospective	731	n/a	n/a	Delayed bleeding-0.7, perforation-0
Yamashina et al. (110)	Prospective	102	76	26	Delayed bleeding-1.96, perforation-0
Rashid et al. (111)	Retrospective	480	19.2	74.4	Intraprocedural bleeding-4.8, delayed bleeding-1.67, perforation-0.21
van Hattem et al. (112)	Prospective	353	0	100	Delayed bleeding-5.1
Zhang et al. (113)	Retrospective	130	92.96	7.04	Bleeding-1.4, perforation-0

En-bloc resection is preferred over piecemeal polypectomy as it allows more accurate histological assessment. In cases of malignant polyps, it gives fundamental information on lateral and vertical margins. Deep submucosal invasion, defined as tumor involvement ≥1 mm (1,000 mm, or SM3 on Kikuchi classification), is associated with a high risk of lymph node metastasis and residual recurrence (10–18%) (114). A meta-analysis of 33 studies showed the overall recurrence risk for EMR resection to be 15% (95% Cl 12–19%). The recurrence rate was higher after piecemeal resection (20%) than en-bloc resection (115). A multicenter prospective study of 1,000 successful EMR procedures for sessile or laterally spreading colonic lesions ≥20 mm in size showed an early recurrent/residual adenoma rate of 16% (95% Cl: 13.6–18.7%) (116). Out of the total, 71.7% of these were diminutive, and 93.1% treated successfully using the endoscopic method. Lesions size >40 mm, use of argon plasma coagulation (APC) for treatment of incomplete polyp resections, and intraprocedural bleeding was identified as risk factors for these recurrent/residual adenomas (116). Consequently, surveillance endoscopy is recommended at 6 and at 16–18 months after piecemeal EMR to detect any recurrence (117). The US Multi-Society Task Force on Colorectal Cancer recommends using adjuvant thermal ablation at the margins of the polypectomy, even when there is no endoscopically visible polypoid tissue for treatment of micropolyp not visible by endoscopy (39). The most common modalities include APC or snare tip soft coagulation. Residual polypoid tissue within the polypectomy site is best treated by avulsing the residual polyp using hot forceps called as hot forceps avulsion technique (39).

Another technique, underwater EMR is also gaining in popularity. Different from conventional injection assisted EMR, where submucosal injection provides a cushion separating the submucosal layer form the muscularis propria (MP), no submucosal injection is performed during underwater EMR to raise the lesion. The polyp is submersed in water and the intraluminal air removed, removing colonic wall tension, and separating the mucosa from the MP. This prevents accidental muscle entrapment with the snare and helps with thermal dissipation, decreasing the risk of perforation during resection. Additional advantages of this technique is that it allows the capture of a larger mucosal surface area in the opened snare, increasing the chance of en-bloc resection without the use of a larger snare, and the resection is faster than compared to the conventional technique. The disadvantage of this technique is bleeding underwater during resection can obscure visualization (118, 119).

### Endoscopic Submucosal Dissection

Endoscopic submucosal dissection (ESD) is an advanced form of polypectomy designed to resect large lesions in an en-bloc manner resulting in lower recurrence rates (120, 121). It was initially described in Japan for resection of early gastric cancer and now adopted to treat complex colorectal polyps (122, 123). ESD allows en-bloc resection of large superficial polyps, especially flat polyps, which would otherwise need piecemeal resection with EMR. A piecemeal resection by EMR lead to increased recurrent rates when compared to en-bloc ESD (124, 125). ESD involves a submucosal injection to achieve adequate submucosal lift and then circumferential incision of mucosa using an endoscopic knife, followed by submucosal dissection underneath the lesion above the muscularis propria (73, 117, 126). ESD is time-consuming, labor-intensive, technically difficult, and has a higher risk of complications like bleeding or perforation (71, 117).

American Gastroenterology Association recommends ESD for colorectal lesions which are too large to ensure en bloc resection with EMR or at higher risk of containing cancer (125). Similarly, the European Society of Gastrointestinal Society recommends that ESD to be considered in patients with colonic and rectal lesions suspected to have a superficial submucosal invasion (sm1 and sm2), which cannot be removed en-bloc by EMR technique (127). Lesions with suspicion for deep submucosal invasion (sm3) or muscualris propria invasion should be referred for surgical management. The greatest benefit of ESD is in rectal lesions. ESD offers a minimally invasive option with adequate R0 resection in selected early rectal cancers (T1,where in cancer is restricted to the submucosa) with no high risk histologic features, ESD has also shown great results in the management of residual/recurrent tumors after EMR, tumors in patients with inflammatory bowel disease, and large colo-rectal polyps (128, 129).

There are 2 different techniques in ESD: the classical technique and the submucosal tunneling technique. The classical technique, with an initial circumferentially dissection around the polyp followed by dissection under the polyp and complete resection of the lesion. The submucosal tunnel leads to the creation of a pocket. The proximal end of the polyp is dissected initially and subsequently, the distal end is raised. The lateral end is not dissected at the beginning to avoid loss of injection fluid and the polyp raise is maintained. An inicision from the distal end is then used to create a tunnel and complete the dissection. Toward the end of the dissection, the lateral walls are dissected (130).

A meta-analysis of 14 studies evaluating the success of en-bloc resection of large colorectal polyps by ESD showed successful en-bloc resection in 84.91% (95% Cl: 77.82–90.82) and complete cure en-bloc resection in 75.39% (95% Cl: 66.69–82.21) (131) (Table 2). Another systematic review and meta-analysis of 97 studies evaluating colorectal lesions resected using the ESD technique showed that the R0 resection rate was 82.9% (95% Cl: 80.4–85.1%) and significantly higher in Asian countries than non-Asian countries (85.6 vs. 71.3%). Similarly, the en-bloc resection rate was 91% (95% Cl 89.2–92.5%), which was also significantly higher in Asian countries than non-Asian countries (93 vs. 81.2%). The complication like recurrence at 12 months (2%), delayed bleeding (2.7%) and perforation (5.2%) were significantly low (132). ESD is an established endoscopic resection method in Asian countries and being slowly adopted in Western countries with increasing practice in Europe over the last decade and now in the United States, mainly in advanced tertiary centers (125).

**Table 2:** Endoscopic submucosal dissection (ESD) for colon polyp studies with more than 100 patients.

** References**	**Study type**	**Number of patients**	**En bloc resection rate (%)**	**R0 resection rate (%)**	**Complications (%)**
**2009–2014**					
Isomoto et al. (133)	Retrospective	278	90.1	79.8	Bleeding-0.7, perforation-8.2
Hotta et al. (134)	Retrospective	120	93	85	Perforation-7.5, bleeding-N.A PMID-21175483
Nimmi et al. (135)	Retrospective	290	90.3	74.5	Post-operative bleeding-1.3 and perforation-4.5
Matsumoto et al. (136)	Retrospective	203	86	86	Bleeding- 0, perforation-7 PMID-20626303
Kuroki et al. (137)	Retrospective	418	98	92	Bleeding-2, 4 perforation-5.26
Toyonaga et al. (138)	Retrospective	268	99	98	Bleeding-0.37, perforation-2.2
Nishiyama et al. (139)	Retrospective	282	89.2	79.1	Bleeding-0.7, perforation-8.1
Saito et al. (140)	Retrospective	1,090	88	89	Postoperative bleeding-1.5, perforation-4.9
Yoshida et al. (141)	Retrospective	250	87	81	Post-operative bleeding-2.4, perforation-6
Byeon et al. (142)	Retrospective	162	87	75	Immediate bleding-1, delayed bleeding-1, and perforation-7.4
Shono et al. (143)	Retrospective	137	89.1	85.4	Perforation-3.6, post-operative hemorrhage-3.6
Sakamoto et al. (144)	Retrospective	101	94	92	Bleeding-0, perforation-1.98
Tamai et al. (145)	Retrospective	614	89.4	87.1	Bleeding 1.4, perforation-2.6
Kiriyama et al. (146)	Retrospective	297	87.2	80.1	Post-procedure bleeding-1.7, perforation- 4.7
Lee et al. (147)	Retrospective	874	97.5	91.2	Perforation-5.3
Nakajima et al. (124)	Prospective	816	94.5	93	Delayed bleeding 2.2, perforation-1.6
Suh et al. (148)	Retrospective	150	98	95.3	Perforations-4.7, delayed bleeding-0
Hori et al. (149)	Prospective	232	93	92	Bleeding-n/a, perforation-2
Nawata et al. (150)	Retrospective	145	99	97	Bleeding-0, perforation-0
Sato et al. (151)	Retrospective	147	94.7	86.8	Bleeding-1.3, perforation-1.3
Sakamoto et al. (152)	Retrospective	164	95	92	Delayed bleeding-3, perforation-4
Takeuchi et al. (153)	Retrospective	816	94	78	Perforation 2.1, bleeding-2.2
**2015–2021**					
Mizushima et al. (154)	Retrospective	122	86.6	87	Delayed bleeding-3.7, perforation-6.7
Tanaka et al. (155)	Retrospective	629	94	92	Bleeding-0.79, perforation-3.1
Yamamoto et al. (156)	Retrospective	107	97.5	91	Bleeding-1.7, perforation-0.8
Hayashi et al. (157)	Retrospective	472	98	87	Bleeding-2.2, perforation-4
Cong et al. (158)	Retrospective	156	83	81	Perforation-2.3, bleeding-3.4
Shigita et al. (159)	Retrospective	222	89.7	83.0	Bleeding-6.3, perforarion-5.4
Sauer et al. (160)	Retrospective	178	88.4	89.4	Delayed bleeding-2.7, perforation-9.3
Youk et al. (161)	Prospective	319	98	80	Perforation-0.6, bleeding-3.1
Spychalski et al. (162)	Prospective	227	79.39	79	Bleeding 4.4, perforation-7.9
Iacopini et al. (163)	Prospective	155	83	71	Delayed bleeding-1, perforation-3
Yamada et al. (164)	Retrospective	423	n/a	81	Delayed bleeding-1 and perforation 3
Boda et al. (165)	Retrospective	1,233	92.6	83.7	Delayed bleeding-3.7, perforation-intraoperative-3.4, and delayed perforation-0.4
Ronnow et al. (166)	Retrospective	301	80	69	Bleeding-3 and perforation-14
Qi et al. (167)	Retrospective	412	99.5	86.9	Bleeding-2.2, perforation-1, post-ESD electrocoagulation syndrome-6.8
Yang et al. (168)	Retrospective	171	82.5	74.9	Bleeding-2.3, perforation-4.1
Tanabe et al. (169)	Prospective	141	91.8	N/A	Delayed bleeding-7.8, perforation-2, post-colorectal ESD coagulation syndrome-4.3
Draganov et al. (170)	Prospective	692	91.5	84.2	Bleeding-2.3 and perforation-2.9

*R0, Radical resection rate; Defined as dysplasia free vertical and lateral resection margins at histology*.

A meta-analysis of 66 studies comparing EMR and ESD for colorectal lesions showed higher en bloc resection rate of 90.5% with ESD compared to 62.8% with EMR (OR 0.18, 95% CI 0.16–0.2) (171). Similar results were reported in other meta-analyses showing higher en bloc resection rates with ESD compared to EMR (99, 172). There are several advancements in endoscopic tools which have made ESD less cumbersome. There are various colonic dissection knives (dual knife, dual-J knife, Hook knife, IT knife, IT-J knife, ERBE knife) and co-agulation grasping forceps for co-agulation of bleeding. However, the traction tools are still lacking making it a challenging procedure (173).

### Hybrid ESD or Knife Assisted Snare Resection

It combines ESD with snaring and thus simplifies the process of submucosal dissection. It is associated with shortening time to perform the procedure and complication rate, although it has lower en-bloc resection rates than typical ESD (174). It involves using an ESD knife to make a circumferential mucosal incision around the lesion, and then the targeted subepithelial lesion is grasped, retracted toward the lumen, followed by snare resection. Resection is aimed for en-bloc removal. This technique uses a standard snare, and needle-knives during ESD (12, 130). It can also be used to resect scarred polyps (recurrence following previous EMR) (12).

Retrospective data was collected from a study in Japan conducted in patients with large colorectal polyps with size >20 mm who underwent either ESD (for 137 lesions in 134 patients) or hybrid ESD (27 lesions in 26 patients). Results showed a shorter procedure time with hybrid ESD (108 ± 59.5 vs. 122 ± 72.2 min) but lower en-bloc resection than the ESD group (66.7 vs. 94.2%). However, there were no significant differences in procedure time, in rates of en bloc resection or complication rates between the two groups (174).

In a meta-analysis, 97 studies evaluated standard technique, and 12 studies evaluated hybrid technique for colorectal lesions suspicious of superficial malignancy showed that R0 and en-bloc resection rate of 60.6 and 68.4%, respectively, for hybrid technique. It was significantly lower than the standard ESD technique with similar adverse event rates (132). Another recent meta-analysis of 16 studies with 751 patients who underwent hybrid ESD for large colorectal lesions showed an en-bloc resection rate and complication rate of 81.63% (95% Cl: 72.07–88.44) and 7.74% (95% Cl 4.78–12.31), respectively. Subgroup analysis of conventional (*N* = 1,703) with hybrid ESD (*N* = 497), procedure time was found to significantly shorter with hybrid ESD (mean difference 18.45 min; *p* = 0.003), lower complication rate (*p* = 0.04), but it has lower en bloc resection rate (*p* < 0.001) (175).

### Endoscopic Full-Thickness Resection

This is another well-established advanced resection technique. The EFTR involves full-thickness plication of the bowel wall secured by an over-the-scope clip followed by bowel wall resection above the clip. Commercially available full-thickness resection device (FTRD^®^, Ovesco, Germany) is a single-step full-thickness device that combines a modified over-the-scope clip with an integrated snare (176). EFTR is for complex polyp that is not amenable to conventional endoscopic resection due to severe fibrosis and scarring, specific anatomical locations (close to a diverticulum or appendiceal orifice), and cases of incomplete resections. Lesions <2.5 cm are suitable for this technique (126, 177). There is a small risk of appendicitis when lesions are resected close to the appendix and some risk of dehiscence due to OVESCO clip falling off the colonic mucosa thereby leading to peritonitis and sepsis. Most of the data is from small studies, so further large, randomized studies are needed, especially compared with other available endoscopic resection techniques (178–181).

## Specimen Handling

Pathological examination of specimens resected by EMR or ESD is a critical step and crucial for diagnosis of lymphatic spread and risk of metastasis. A clinical report with endoscopic information and a pinned formalin-fixed specimen with margins properly oriented by an endoscopist are necessary to start pathologic assessment (182, 183). The specimens are pinned onto a paraffin wax block and submerged in formaldehyde before submitting for the pathologic assessment to preserve tissue shape, size, and orientation. Knowledge about the appearance of the lesion is required to have the orientation of the specimen. To help orientation of en bloc resection specimens, these specimens are first flattened and fixed at their periphery with thin needles before immersion to formalin. The distance of cancerous tissue from the resection margin should be included for pedunculated specimens. Similarly, non-pedunculated cancerous lesion specimens should include the histology, depth of the lesion, cancerous involvement of the lateral and vertical margins, presence of tumor budding, degree of pathologic differentiation, and lymphatic and blood vessel involvement (39).

## Complications

These advanced endoscopic techniques for the removal of complex polyps have an increased risk of various complications. Bleeding and perforation are two main complications associated with EMR and ESD procedures. Other complications include non-specific postprocedural pain, post polypectomy syndrome, residual tissue. It is very important for the endoscopists to prevent, early recognition and prompt management of these complications (Tables 1, 2).

### Bleeding

Bleeding is the most common complication after the EMR procedure, reported in 0.7–24% of the cases. It can be classified into immediate post-polypectomy–IPPB (intraprocedural) or delayed post-polypectomy–DPPB (post-procedural) bleed (184). Intraprocedural bleeding has been reported in 11–22% of cases, and it can be controlled endoscopically, but it does prolong the procedure (72, 83, 185). The risk factor for intraprocedural bleeding includes large polyps, tubulovillous or villous lesion, minimally elevated sessile polyps, limited operator experience with EMR. This bleeding is effectively managed during the procedure using snare tip soft coagulation, coagulation grasping forceps, or endoclips (72, 186). Postprocedural bleeding occurs hours to days after the procedure, and the rate of bleeding has been reported between 2 and 11%, with clinically significant bleeding in 6% of the cases (72, 83, 185). Risk factor for delayed bleeding includes lesions in the right colon, large lesions with size ≥40 mm, age more than 75 years, antiplatelets or anticoagulants within seven days of procedure and intraprocedural bleeding (83, 187–189).

The bleeding rate after ESD ranges from 0 to 11.9% for upto 15 days post procedure. It can be classified into immediate (intraprocedural) or delayed (post-procedural) bleed (190, 191). A recent meta-analysis of 104 studies showed the rate of immediate and delayed major bleeding of 0.75% (95% Cl: 0.31–1.8%) and 2.1% (95% Cl: 1.6–2.6%), respectively, after ESD for colorectal lesions (192). Risk factors for delayed bleeding include the lesion's size, sessile type, the occurrence of intraprocedural bleeding, use of prior anti-thrombotic agents (193, 194). Recent studies have shown lesions in the cecum and rectum have a higher incidence of delayed bleeding after ESD (193, 195, 196).

Several randomized studies have evaluated the utility of clip closure after resection of large non-pedunculated colonic polyps (197–199). Results of these studies argue against the routine use of prophylactic clip placement after polypectomy. However, clip closure is recommended to prevent DPPB after resection of large colorectal lesion ≥20 mm in size and proximal to the splenic flexure (200). Closure of lesion ≥20 mm is further supported by a recent meta-analysis of 13 studies that showed that prophylactic clipping (1.4%) was associated with a lower rate of delayed bleeding compared to no clipping (5.2%) (pooled OR:0.24, 95% Cl: 0.12–0.50) after the EMR procedure (201).

### Perforation

Another potential complication after EMR and ESD is colonic perforation. The risk of perforation is low after EMR, with the reported risk of 1–2%. In a meta-analysis of 50 studies, endoscopic perforation occurred in 1.5% (95% Cl: 1.2–1.7%) of cases following EMR for colorectal polyps ≥20 mm (86). Risk factors include using larger diameters snares (≥20 mm), proximal location, bulky lesions, and cutting current. Perforation is more common following colorectal ESD, and the rate reported to be up to 3.3 to 10% (140, 171, 172, 202–204). A meta-analysis of 66 studies comparing EMR and ESD for colorectal lesions, perforation rate was found to higher with ESD compared to EMR (4.8 vs. 0.9%, *p* < 0.0001) (171). Similar results were reported in other meta-analyses showing higher perforation risk with ESD compared to EMR (99, 172). A meta-analysis of 97 studies with colorectal lesions removed by standard ESD showed a perforation rate of 5.2% (95% Cl: 4.4–6.1%). This meta-analysis also included 12 studies with colorectal lesions removed by hybrid ESD and showed a perforation rate of 4.8% (95% Cl: 2.4–9.1%) (132). Risk factors for perforations during ESD include tumor size, location, submucosal fibrosis, and operators with limited experience (205, 206). Perforations are more in the ascending colon and cecum due to its thin wall (207, 208).

Deep muscle injury without overt perforations (Sydney classification Type 2–3) or small perforations (up to 10 mm) recognized during colonoscopy can be managed endoscopically with through the scope clips. Surgery can be avoided for overt perforations (Type 4–5) up to 30mm by using larger capacity over the scope clips (Ovesco^®^, Endoscopy AG, Tübingen, Germany, or the Padlock Clip^®^ Defect Closure System, Steris HC, OH, USA); however, it requires surgical intervention if recognized late or if there is overt contamination (83, 171, 190, 209, 210). In selected cases, endoscopic suturing devices (Overstitch Endoscopic Suturing System™, Austin, Texas, USA), which provide full thickness closure, have been used to close larger lesions (211).

### Post-polypectomy Syndrome

Post polypectomy syndrome is an electrocoagulation injury to the bowel wall after endoscopic treatments, including conventional polypectomy, EMR, and ESD. Injury to the wall induces a transmural burn and localized peritonitis, which in turn causes serosal inflammation (212, 213). Incidence of post polypectomy syndrome varies from 1% after conventional polypectomy or EMR to 9% after ESD (212). The patient presents with abdominal pain, fever, tenderness, leukocytosis, elevated C-reactive protein after an endoscopic procedure like polypectomy, ESD, or EMR, without any obvious perforation on abdominal imaging like radiograph or computed tomography (138, 190, 213). Most of these patients are successfully managed with conservative treatment, including bowel rest, broad-spectrum antibiotics, and hydration. Patients should be reevaluated for possible delayed perforation in case they are not showing improvement or getting worse with conservative management (212, 214, 215).

### Stenosis

Post-ESD stenosis is defined as narrowing through which a standard endoscope cannot be advanced (130). Fortunately, there are only a few studies describing post ESD stenosis after colorectal ESD. This is mostly seen when more than 75% of the circumferential lesion is resected. In a retrospective study of 822 patients who underwent colorectal ESD, 0.49% (4/822) of patients developed stenosis post-procedure. Post-ESD stenosis occurred in 11.1% of patients who underwent circumferential resection between ≥90 and 100%, and in 50% of patients who underwent 100% circumferential resection (216). Similarly, in another study of 69 patients with large rectal neoplasm that required ≥75 % circumferential resection, 19.7% of the patients developed post-ESD rectal strictures. In the subgroup analysis, patients who underwent total circumferential ESD developed stricture in 71.4% of cases, and those who underwent ≥90% circumferential resection developed stricture in 43.8% of cases (217). These studies showed that ≥90% circumferential resection is a risk factor for stenosis after colorectal ESD. Most of these patients are managed by endoscopic balloon dilatation (130, 216, 217).

## Future Direction

The main challenges in performing ESD in the west have been higher prevalence of colorectal polyp requiring ESD, unlike in Japan where ESD is performed more in the stomach. There is more prevalence of obesity in the west, which makes the colon tortuous and thereby procedure technically challenging. The risk of procedure complications are higher due to thin colonic wall unlike the thick gastric wall. Therefore, there is a need for more advanced tools for polyp traction and post polypectomy defect closure to safely perform the procedure. In US, although there is increase interest in ESD for colorectal lesions, the adoption has been slow due to lack of dedicated training in ESD.

One of the common traction approaches is the distal attachment (cap) attached at the endoscope's end, which helps move the lesion away and allows visualization of the dissection plane (12, 218). Various traction devices have been developed to facilitate faster ESD with a lower complication rate (12, 219). One simple method to achieve traction is to have a silk line (like a dental floss) tied to a hemostatic clip to the edge of the lesion and pulling the lesion proximally using the line away from the colonic wall to perform a safe dissection. It is a simple method; it does not require any novel equipment but requires the endoscope's reinsertion (220). Internal traction modifies the above method by attaching a micro-tech elastic band or ring, or nylon to a clip attached to the lesion and another clip to the opposite end. No reinsertion of the endoscope is required (221, 222). Another novel system consisting of an expandable working chamber with two independent instrument guides (LIG) has been used in the *in vivo* model to achieve safe and effective completion of ESD and submuscular dissection by improving visualization, access to the target tissue, and improving procedure time (223).

Another technique, thin endoscope-assisted ESD, allows traction in any direction where the second endoscope is inserted alongside the main endoscope. At present, this technique is limited to the distal sigmoid colon and rectum (224). Other techniques like a three-dimensional printed overtube system with two manipulator arms at the tip and magnetic traction methods have shown promising results in animal models (225, 226). Most of these techniques are not in mainstream use. Clip and string are commonly used in most ESD practices as they don't require any special equipment (12).

Post polypectomy defect closure post resection is another significant challenge especially in the right side colon. Di-Lumen or Lumendi is an accessory to the endoscope, which works like an overtube. This helps in reducing the loop in the colon thereby ensuring better stability with right side colon polyp resection and faster access to the lesion especially in the right side of the colon. The time for resection of large polyps in the right side of the colon has decreased by nearly 50% due to Lumendi. The overtube can then be used as a conduit to pass the Apollo overstitch. The overstitch can usually only reach the left side of the colon, but because of the reduced loop and the colon being less tortuous and straight, it is now able to reach the right side of the colon for safe closure of the post polypectomy defect. The disadvantage in using an apollo overstitch is that the scope has to be removed, the suture has to be loaded and the scope again reinserted which can add to the already prolonged procedure time (227, 228). A novel suture device called endoscopic tack is now FDA approved and the post polypectomy defect can be safely closed without scope removal (229). In addition to the current colonic dissection knives, there is a new speed boat Knife (Creo Medical) which can help in simultaneous injection, dissection, and coagulation. This helps in speedy dissection and en-bloc resection (230).

## Conclusion

Management strategies for complex polyp have evolved immensely over the last two decades and continue to do so. This is due to a better understanding of complex polyps' pathophysiology and advancement in technology, which led to the development of novel endoscopic tools and techniques and more effective management of complications. Whenever an endoscopist encounters a complex colorectal lesion, many patient-specific variables like age, comorbidities, use of anticoagulants and polyp-specific like lesion size, location, and malignancy risk should be considered before deciding to either resect or refer to an advanced endoscopist. Most premalignant lesions can be removed with advanced endoscopist techniques, but these procedures require an endoscopic expert in the field, a center with the appropriate equipment, and trained staff. Management of complex polyp with advanced endoscopic techniques like EMR, ESD, and hybrid approcahes will lead to decreased morbidity, mortality, and healthcare cost by decreasing the need for surgical interventions. This will prevent unnecessary morbid surgical procedures for benign lesions.

## Author Contributions

RM, MG, CU, AP, HG, and JE: conception and design. RM, MG, and JE: literature search. RM and MG: first draft. All authors critical revision, editing, and final approval.

## Conflict of Interest

The authors declare that the research was conducted in the absence of any commercial or financial relationships that could be construed as a potential conflict of interest.

## Publisher's Note

All claims expressed in this article are solely those of the authors and do not necessarily represent those of their affiliated organizations, or those of the publisher, the editors and the reviewers. Any product that may be evaluated in this article, or claim that may be made by its manufacturer, is not guaranteed or endorsed by the publisher.
